# Animal Welfare Assessment in Antibiotic-Free and Conventional Broiler Chicken

**DOI:** 10.3390/ani11102822

**Published:** 2021-09-27

**Authors:** Luigi Iannetti, Sara Romagnoli, Giuseppe Cotturone, Michele Podaliri Vulpiani

**Affiliations:** OIE Collaborating Centre for Animal Welfare, Istituto Zooprofilattico Sperimentale dell’Abruzzo e del Molise “G. Caporale”, Via Campo Boario, 64100 Teramo, Italy; romagnolisara.vet@gmail.com (S.R.); veterinariocotturone@gmail.com (G.C.); m.podaliri@izs.it (M.P.V.)

**Keywords:** antibiotic free, animal welfare, broiler

## Abstract

**Simple Summary:**

Antibiotic resistance in the veterinary field, other than making the control of infectious diseases in farm animals progressively more difficult, can increase the risk that resistant microorganisms are transferred from animals to humans either directly—by contact or from food of animal origin—or indirectly due to environmental contamination. The poultry sector is now moving towards antibiotic-free production in order to meet the rising market demand, but this could affect the health and welfare of chickens. In this study, we compared the welfare of broiler chickens raised with and without the use antibiotics on a commercial scale. We found no correlation between the absence of antibiotics and poor animal health. There is no necessary correlation between the absence of antibiotics at farms and poor health of the animals, given that adequate animal-welfare-friendly management tools and methodologies are in place. These should be, however, adequately standardised in specific guidelines. In this way, it will be possible to reduce the dependence of the livestock sector on antimicrobials with regard to animal welfare and human health.

**Abstract:**

The poultry sector is moving towards antibiotic-free production, both to challenge the increasing spread of the antibiotic resistance phenomenon and to meet market demands. This could negatively impact the health and welfare of the animals. In this study, the welfare of 14 batches of 41–47-day-old broilers raised by the same integrated company with and without antibiotics was assessed using the Welfare Quality^®^ protocol. The total welfare score did not significantly differ between the two systems: the good-feeding principle was, on average, higher in the conventional batches, with statistical significance (*t* = −2.45; *p* = 0.024), while the other welfare principles (good housing, good health and appropriate behaviour) were slightly better in the antibiotic-free batches. Despite stocking densities averagely higher in the antibiotic-free batches, the absence of antibiotics did not seem to impact the good-health principle; in particular, hock burns, foot pad dermatitis and lameness were significantly less severe in the antibiotic-free batches (*p* < 0.0001, *p* = 0.018, *p* < 0.0001, respectively), which showed also a lower death rate (2.34% vs. 2.50%). Better management of antibiotic-free batches was reported, particularly concerning litter conditions. Further studies would be required to identify and standardise a set of managerial methodologies in order to improve the health of broilers raised without antibiotics.

## 1. Introduction

In the European Union, over 7.2 billion broiler chickens are slaughtered every year for the production of around 13 million tonnes of meat [1]. There is scientific evidence that intensive farming for poultry meat production can result in serious animal welfare issues [2]. Animals are genetically selected to have a rapid growth rate during their short life (5–7 weeks) in large poultry houses with a high density of animals. These conditions can create more challenges in avoiding increased moisture in the litter, higher temperatures and increased levels of ammonia, often resulting in serious health and welfare problems in the animals, with frequent pathologies such as gait problems, lameness and cardiovascular and respiratory diseases [3]. 

Animal infections sustained by multidrug-resistant zoonotic pathogens, such as *Salmonella* and *Campylobacter*, which are highly frequent in broiler chicken farms, represent a potential risk for human health. According to the last report of the European Food Safety Authority, the proportion of positive broiler flocks in the European Union is 13% for *Campylobacter* and 3.63% for *Salmonella* [4]. Moreover, in recent decades, the excessive use of antibiotics in both human and zootechnical fields has increased the spread of the antimicrobial resistance (AMR) phenomenon. The use of antibiotics in animal production worldwide is estimated at around 240,000 tonnes/year [5]. Evidence suggests that there is a link between the use of antimicrobials in veterinary medicine and the spread of antimicrobial resistance in pathogens that infect humans [6]. Increasing antimicrobial resistance also hinders disease control in farm animals and increases the risk of direct or indirect transmission of resistant pathogens to humans [7]. Therefore, reducing antimicrobial use in agriculture is vital for human and animal health. In fact, the spread of the phenomenon in the veterinary field, in addition to making the control of infectious diseases in farm animals increasingly difficult, can increase the risk that resistant microorganisms are transferred from animals to humans either directly—by contact or from food of animal origin—or indirectly due to environmental contamination. Even the transmission from animals and foodstuffs of non-pathogenic microbes to humans can result in the spread of the genes of antibiotic resistance, which can be easily transferred from the veterinary sector to human healthcare. This can happen, for example, through the well-known mechanism of resistance plasmid transmission, which can happen fast between cells of not only similar but also different species of bacteria [8]. 

According to the global strategy on antibiotic resistance [9], the World Organisation for Animal Health encourages the search for alternative systems to the use of antibiotics, including the study of animal-welfare-friendly management tools and methodologies that, with regard to the welfare of farm animals, could reduce the dependence of the livestock sector on antimicrobials. Regulatory interventions and practical strategies that recommend the rationalisation of the use of antibiotics should lead to a significant decrease in their use. It is well known that well-cared-for and adequately housed animals are less prone to infections and require fewer antibiotics [10].

Several companies in the poultry sector are moving towards the production of specific lines of products obtained from animals raised without the use of antibiotics, especially to meet market demands: consumers are increasingly sensitive to the problem of antibiotic resistance, and in any case, they identify the products obtained without the use of antibiotics as “healthier” and also more “animal-welfare-friendly” [11]. Studies have instead found that the growing trend of raising broilers without antibiotics could negatively impact the health and welfare of the animals [12], particularly increasing disease incidence and mortality [13,14]. In fact, the reduction in the use of antibiotics should be a gradual and controlled process, which should take into account the effective welfare of the animals raised; the absence of medical care for the animals may have a negative impact on the quality of life of the animals, if not accompanied by adequate additional structural and managerial interventions. 

This study, carried out in the framework of a wider project funded by the Italian Ministry of Health for the study of antibiotic resistance in the poultry sector, aimed to investigate the effective welfare of broiler chickens raised by the same integrated company with and without the use of antibiotics. The Welfare Quality^®^ protocol for the welfare assessment of broiler chickens [15] was used in batches from the two raising systems, and the results were compared and analysed with regard both to the total welfare score and to the scores given by the four different welfare principles included in the protocol (good feeding, good housing, good health and appropriate behaviour).

## 2. Materials and Methods

### 2.1. Animals

Fourteen batches of broiler chickens from 6 farms located in the Abruzzi region (central Italy) were included in the study, which was carried out during a 9-month period. All belonged to the same integrated poultry company that manages the production chain at all levels, from hatchlings to slaughtering. A batch meant a flock of broiler chickens with identical characteristics (age and genotype), introduced in the same poultry house at the same time, raised under the same conditions and slaughtered on the same day. A farm meant one premise of the poultry company where different batches of broilers were raised, including different multi-storey buildings (two or three floors), each containing a variable number of poultry houses. A poultry house or house meant the wide area where each batch was raised, consisting usually of half of each building’s floor. The environment of each house (temperature and humidity) was electronically controlled with a forced ventilation system. The introduction of new batches of broilers in a house always followed an all-in, all-out system, with a period of a 2–3-week break between one production cycle and the other. 

For each entry into the farm (7 entrances in all), two batches were examined on the same day, one of which was raised without the use of antibiotics (antibiotic-free), while the other was raised in a conventional manner, including the use of antibiotics. Diet was fully vegetarian in the antibiotic-free batches, without the presence of genetically modified organisms (GMO-free), while proteins of animal origin were included in the diet of broilers raised in a conventional manner.

Batches were identified with a letter indicating the farm (from A to F), followed by another letter indicating the type of management (A: antibiotic free; C: conventional) and finally a number indicating the batch (batch 1 or batch 2 for that farm). Two batches per farm (one antibiotic free and one conventional) were examined, except farm A, for which four batches were tested (two antibiotic free and two conventional). Therefore, in total, 7 pairs of antibiotic-free/conventional batches were evaluated, in 6 different farms.

All the batches were slaughtered when they were from 42 to 48 days old, precisely 42 days for the batch pair BC1 and BA1, 46 days for the pair DC1 and DA1 and 48 days for all the other pairs. The genotype was Ross 308 for all batches; the number of animals per batch at the beginning of the rearing cycle ranged from 9000 to 41,700. The area of the poultry houses where batches were kept ranged from 675 to 2160 m^2^. The male:female ratio was about 2:1. The total number of animals present in the whole farm ranged from 120,000 to 400,000. The animals were all slaughtered in the same slaughterhouse, also located in the Abruzzi region less than 15 km from all the farms. 

### 2.2. Animal Welfare Assessment

The day before slaughtering, each batch underwent animal welfare evaluation with the Welfare Quality^®^ protocol for broilers [15], modified according to De Jong et al. [16], in order to make data collection faster. Each pair of antibiotic-free/conventional batches was evaluated on the same day and therefore at the same age, which was always the day before slaughter, therefore ranging from 41 to 47 days old. Evaluations were carried out across a 9-month period, from October 2018 to July 2019. According to the protocol, data were mostly collected on the farms and then completed at the slaughterhouses with information about diseases and lesions reported from carcass inspection. The protocol was produced as part of the 6th Framework Research programme of the European Commission, and it is based on the internationally recognised five freedoms, which according to the World Organisation for Animal Health (OIE) should provide valuable guidance regarding animal welfare [17]. The animal welfare measures used in the protocol were generated from four animal welfare principles (good feeding, good housing, good health and appropriate behaviour) and further divided into 12 criteria, 9 of which were applied in the conditions of this study and were therefore evaluated. In Table 1 are detailed the criteria and relative measures that were considered for the animal welfare assessment. These were both animal based (observations of the response of the animal to the environment and management) and resource based (evaluation of the premises and the environment where the animals were kept and evaluation of their management). The evaluation of each batch required about 2 h and produced a score for each animal welfare principle; a general animal welfare score (total welfare score) was then calculated according to Tuyttens et al. [18] and assigned to each batch. This score was composed of the sum of the scores assigned to each of the four animal welfare principles (maximum score 100) and ranged from 0 to 400. Batches were also classified according to the categories defined by the Welfare Quality^®^ protocol, namely excellent (score more than 55 on all principles and more of 80 in two of them), enhanced (more than 20 on all principles and more than 55 in two of them), acceptable (more than 10 on all principles and more than 20 in three of them) and not classified (batches that did not meet the minimum acceptable standards).

### 2.3. Data Analysis

Collected data were exported to Microsoft Excel version 2010 (Microsoft, Richmond, VA, USA) and then analysed with the statistical software XLstat (Addinsoft, Belmont, VA, USA) to verify the presence of statistically significant differences between the antibiotic-free and conventional batches. The analysis was carried out considering both the total welfare score and the four principles that composed it (good feeding, good housing, good health and appropriate behaviour), individually considered. Given that the evaluations were all carried out by pairs of batches (conventional vs. antibiotic free), and given a normal distribution of the results, a paired *t*-test for dependent means was used, with significance set at *p* < 0.05. It should be acknowledged that two pairs of batches were raised on the same farm, while the others were all raised on different farms. In any case, this should not have biased our results, as all the pairs of batches were from the same integrated poultry company, where structural differences between different farms were quite limited.

## 3. Results

### 3.1. Batches’ Characteristics

In all, 14 batches of broilers were subjected to animal welfare assessment with the Welfare Quality^®^ protocol, at 6 different farms, of which 7 batches were raised conventionally and 7 without the use of antibiotics. In addition, 10 batches out of 14 (71.4%) had been subjected to thinning (reduction in the number of animals) around the 32nd–35th day of rearing, when usually all the female individuals were removed to be slaughtered for the production of rotisserie chicken. In any case, when a conventional batch was subjected to thinning, so was also the correspondently paired antibiotic-free batch. The number of animals per batch at the moment of the welfare evaluation (day before slaughtering) ranged from 4306 to 28,976. The stocking density on the day of slaughtering ranged from 20 to 38 kg/m^2^ in the 10 batches that had been subjected to thinning and from 37 to 46 kg/m^2^ in the other batches. The stocking density was averagely higher in the antibiotic-free batches (36.4 kg/m^2^ vs. 34.1 kg/m^2^). The breeding facilities, all consisting of multi-storey sheds with forced ventilation, showed a controlled temperature of about 18 °C, feeding and automatic watering. The photoperiod was set to ensure at least 6 h of dark per day.

### 3.2. Welfare Quality Scores

**Figure 1:**
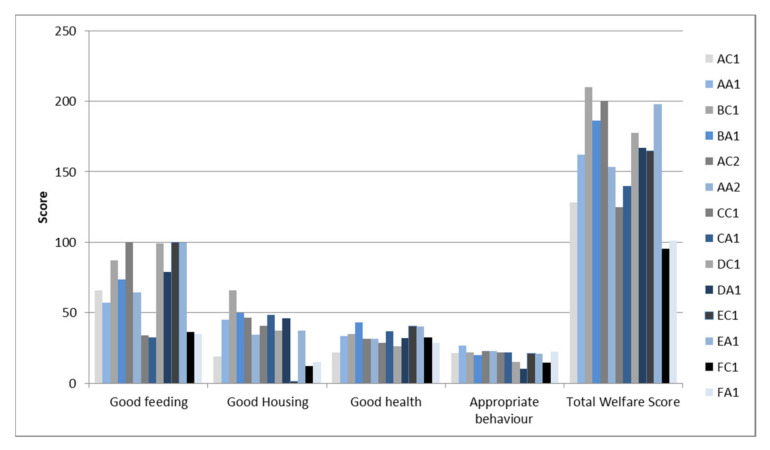
Scores assigned to each batch using the Welfare Quality^®^ protocol. The results of the four principles (good feeding, good housing, good health and appropriate behavior) and the total welfare score of each of the 14 batches are displayed. Pairs of batches evaluated on the same day (conventional vs. antibiotic free) are shown near each other. The conventional batches are in the grey color scale, and the antibiotic-free batches are in the blue color scale.

**Figure 2:**
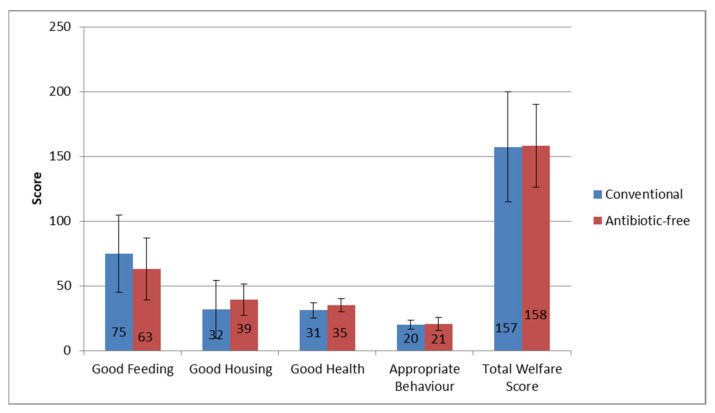
Mean scores assigned to each of the four welfare principles and average total welfare score calculated in conventional (blue) and antibiotic-free (red) batches. Standard deviation bars are reported.

Batches were firstly classified according to the categories given by the Welfare Quality^®^ protocol. Only one batch, raised with the conventional method, was categorised as enhanced (batch BC1), and two batches, both conventional (EC1 and FC1), were classified as unacceptable (not classified). All the other 11 batches (4 conventional and 7 antibiotic free) were classified as Acceptable. A total welfare score was then calculated for each batch by adding up the scores from the four principles, and it ranged from a minimum of 95 in the conventional batch FC1 to a maximum of 210 in the conventional batch BC1. In Figure 1 are detailed the results obtained for each batch, including each welfare principle and the total welfare score. A comparison between the average scores reported in the two groups (conventional vs. antibiotic free) is shown in Figure 2. 

### 3.3. Welfare Principle Analysis

Statistical analysis highlighted the absence of statistically significant differences between the pairs of batches (conventional batches vs. antibiotic-free batches) with regard to the general level of the total welfare score (*t* = 0.074; *p* = 0.477; not significant). Considering the individual principles, the good-feeding principle was, on average, higher in the conventional batches (75 vs. 63), with statistical significance (*t* = −2.45; *p* = 0.024). This could be linked to an averagely higher stocking density highlighted in the antibiotic-free batches (36.4 kg/m^2^ vs. 34.1 kg/m^2^). The other welfare principles (good housing, good health and appropriate behavior) were always slightly better for the antibiotic-free batches, albeit without statistical significance. In particular, the good-health principle scored higher on average in the antibiotic-free batches (39.4 vs. 31.8), bordering on statistical significance (*t* = 1.88; *p* = 0.054). 

Concerning indicators included in these principles, particular attention was paid to the ones included in the good-health principle, given that this is considered one of the main potential issues linked to antibiotic-free broiler farming. In Table 2 are shown the results of the mean scores assigned for hock burns, footpad dermatitis and lameness evaluations in comparison between the antibiotic-free and conventional systems. The scores of these conditions were averagely lower (e.g., better, given a range from 0 = no symptoms to 2 = severe symptoms) in the antibiotic-free batches, with statistically significant difference. 

The death rate ranged from 1.23% in antibiotic-free batch CA1 and conventional batch CC1 to 3.90% in conventional batch FC1, and it was averagely lower in the antibiotic-free batches (2.34% vs. 2.50%), albeit without a statistically significant difference between the two categories (*t* = 0.3613; *p* = 0.7242; not significant). The death rate (mortality due to uncontrolled deaths, not including culling) in the different batches is detailed in Table 3.

With regard to the quality of litter, which was one of good housing indicators, it was also averagely higher in the antibiotic-free batches (average score of 31.1 vs. 20.4). In particular, its value scored higher in the antibiotic-free batches in four pairs out of seven, was equal in two pairs and scored higher in the conventional batch in only one pair. 

## 4. Discussion

In this study, a comprehensive protocol (Welfare Quality^®^) was used to evaluate all aspects of broiler welfare in farms in order to spot the presence of differences between the batches of animals included in the project, with particular reference to conventional or antibiotic-free farming methods. These batches were grown according to similar standards, given that they were all from the same integrated poultry company that directly managed all stages of the poultry meat production line, from hatchlings to slaughtering. Moreover, the comparison between the two management methods was always made using pairs of batches raised on the same farm (different poultry houses) and in the same period, with the only difference in the antibiotic management system, therefore reducing to a minimum all the other possible variables. 

Currently, the antibiotic-free management type is increasingly preferred by various producers based on pressing market demands. The use of farming systems that do not include the use of antibiotics could, potentially, have an impact on the health and, therefore, on the welfare of the animals raised, as certain diseases such as coccidiosis and necrotic enteritis could be difficult to combat without using antimicrobials. However, the World Organisation for Animal Health [9] considers the reduction in the use of antimicrobials, the contrast of antibiotic resistance and animal welfare as three closely related factors in a one-health perspective; in fact, the development of diseases that require the use of antibiotics may be closely related to the use of intensive farming systems characterised by high animal densities and that are detrimental to the level of welfare of the animals themselves. Recent studies have investigated these topics, driven from the hypothesis that broilers never given antibiotics could have a higher likelihood of disease. In fact, according to a survey carried out in the U.S., most producers and veterinarians have “concerns about negative impacts to animal health and welfare” for animals raised without the use of antibiotics, and they also declare that their involvement in this kind of farming is usually due to “fulfilling a client/customer request” [11]. Karavolias et al. [12] found that broilers raised without antibiotics have a greater incidence of three specific health conditions, namely eye burns, footpad lesions and aerosacculitis. However, these results were admittedly potentially biased by the fact that data were not sourced from a specific experiment but from the analysis of a dataset extracted from a wide data management system, including different locations and companies of different sizes. Moreover, it should also be considered that the potentially negative absence of antibiotics could be, at least partially, compensated by an improvement in other managerial factors, which could noticeably vary from one company to another and even from one farm to another. In addition, in our study, even if conducted in the same geographical area (all farms were within a 15 km area) and in the same integrated poultry company, a certain variability was highlighted, with regard to the general level of animal welfare, between the different batches, as evidenced in Figure 1. This variability was particularly marked among the different batches reared with the conventional method; in fact, both the batch with the highest score (BC1, enhanced) and the two batches classified as unacceptable, with the lowest scores (EC1 and FC1, not classified), were all reared with the conventional method. On the contrary, the batches raised with the antibiotic-free system showed greater uniformity concerning the total welfare score. In any case, no statistically significant differences were reported between the two management systems with regard to the total welfare score, but only considering individual principles, and particularly the good-feeding principle, which was significantly higher in conventional batches, probably as a consequence of the higher stocking density reported in the antibiotic-free batches. Our finding of a usually higher stocking density is actually the opposite of what was previously reported, considering that it is commonly believed that antibiotic-free broiler production should be typically associated with additional management strategies such as reducing the stocking density to alleviate negative effects related to the absence of some medical treatments [14,19]. In our case, instead, the poultry company seemed to not want to renounce the income of a high-valued product, therefore preferring to act over other management strategies rather than lowering the stocking density, such as the quality of the litter that was usually better in antibiotic-free batches. This approach could be justified as it has been reported that considering stocking densities between 27 and 39 kg/m^2^, at least feeding and drinking times should not change [14]. However, according to the Welfare Quality^®^ protocol, which bases its score both on the number of drinking points in relation to the number of chickens and on the quality of the carcasses at slaughterhouse, the good-feeding capacity of the animals should be affected, even if in our study the mean stocking density in both antibiotic-free and conventional batches was within this range (36.4 kg/m^2^ vs. 34.1 kg/m^2^). It is interesting to note that according to our results, the absence of antibiotics did not seem to impact the good-health principle that scored at the minimum in a conventional batch (24.1) and at the maximum in an antibiotic-free batch (44.7). These observations were confirmed by the statistical analysis, which showed that the good-health principle was even better on average in the antibiotic-free batches, with a similar average death rate, without statistically significant differences between the two types of farming (2.50% in conventional, 2.34% in antibiotic free). Considering the above-mentioned results, it should be highlighted that the Welfare Quality^®^ protocol, despite being the most comprehensive and elaborate protocol for holistic assessment of broilers’ welfare currently available, is subjected to a number of limitations that have been reported by different authors during the last decade. For example, it was noted that its ability to discriminate between flocks is strongly influenced by a few measures such as drinker space and stocking density, which should be more considered as risk factors for poor welfare rather than as animal-based outcome measures [20]. Moreover, other measures used in the protocol have shown limitations, such as the avoidance distance test [21] and the qualitative behavioural assessment [22], which actually produce the appropriate-behaviour principle score. For these reasons, in this study, some results from single-animal welfare measures, deemed as more relevant, were also individually analysed. In particular, the most frequent health issues in intensive poultry farms, such as hock burns, foot pad dermatitis and lameness scored averagely better in antibiotic-free batches, despite the higher stocking density. As reported in Table 2, statistically significant difference was highlighted between the two systems for all the three locomotor problems considered, with high significance particularly for hock burns and lameness (*p* < 0.0001). 

As shown in Table 3, the death rate was similar in the two systems, ranging from 1.23% in both antibiotic-free and conventional batches of farm C to 3.90% in the conventional batch of farm F, and therefore was probably related to the general management of the farm rather than to the use or not of antibiotics. The maximum difference in the death rate between batches raised with the two different systems but on the same farm F was 1.33%, with the highest rate in the conventional batch (3.90% in the conventional batch vs. 2.57% in the antibiotic-free batch). Moreover, in another farm (B) characterised by rather high mortality, the situation was reversed, with higher mortality in the antibiotic-free batch (3.27% vs. 2.07%). 

According to the literature, there are many factors influencing the occurrence and severity of foot pad dermatitis and other foot lesions in broiler chickens, including stocking density, characteristics of the birds, housing conditions, weather and thinning of flocks [23,24]. However, litter quality and, in particular, the occurrence of wet litter is commonly considered the most important one [25,26]. In this study, better litter quality was commonly reported in the antibiotic-free batches, and this could explain the lower severity of locomotor problems in those batches. It would be interesting to know the differences in litter management used in the two categories, given that litter quality was better in the antibiotic-free batches even in the presence of higher stocking density. The diet used was different between the two bird categories, and in particular it was totally of vegetable origin in the antibiotic-free batches, but unfortunately, its detailed composition was not investigated. That could be the objective of further research, as it has been reported that particularly dietary protein (protein concentration and source) can directly affect litter quality [26]. More factors could also be studied, in particular the role of litter materials and the possible indirect effects that the misuse of antibiotics could have on the quality of litter, e.g., through impacting the gut microbiota and, therefore, the characteristics of faeces [27]. However, the results of this study highlight that there is no necessary correlation between the absence of the use of antibiotics in farms and the poor health of the animals, given that adequate management aimed to improve specific farming conditions is in place. In general, further studies would be needed to identify and standardise the characteristics of a set of managerial factors that should be considered in order to improve the health of broilers raised without the use of antibiotics.

## 5. Conclusions

In conclusion, this research highlighted the presence of differences in the approach to antibiotic-free farming in the various batches examined, with various levels of welfare detected in the farms despite all being part of the same integrated poultry company. The approach to the antibiotic-free system seemed, in general, to be strongly driven by the market demand, with stocking densities averagely higher than conventional. Despite that, the remaining management conditions, litter quality in particular, were usually better in the antibiotic-free batches, and this was probably the determining factor that allowed ensuring for the animals a satisfactory state of health and welfare. Overall, the results obtained highlight the need for the standardisation of broiler-farming methods without the use of antibiotics, possibly through specific regulatory guidance that can address producers in this increasingly popular system of farming broiler chickens in order to protect the welfare of farmed animals and the health of consumers.

## Figures and Tables

**Table 1:** List of the principles, criteria and measures that were assessed for animal welfare evaluation in each batch according to the Welfare Quality^®^ protocol for poultry (broilers).

Principles	Criteria	Measures (Score Range or Measure Unit)	No. of Birds Tested per Batch
Good feeding	Absence of prolonged thirst	Drinker space (drinkers/birds)	Whole batch
	Absence of prolonged hunger	Emaciated carcasses at slaughterhouse (%)	Whole batch
Good housing	Comfort around resting	Plumage cleanliness (0–3), litter quality (0–4), dust sheet test (0–2)	100, 5 locations, whole batch
	Thermal comfort	Panting, huddling (%)	100
	Ease of movements	Stocking density (kg/m^2^)	Whole batch
Good health	Absence of injuries	Lameness (gait score 0–2), hock burns (0–2), foot pad dermatitis (0–2)	125, 100, 100
	Absence of diseases	On-farm mortality, culls on farm, carcasses at slaughterhouse with signs of disease (%)	Whole batch
Appropriate behaviour	Good human–animal relationship	Avoidance distance test (no. of touched animals)	21 locations
	Positive emotional state	Qualitative behaviour assessment (QBA)	Observations at 1–8 points

**Table 2:** Comparison of the average scores obtained for hock burns (HB), foot pad dermatitis (FDB) and lameness evaluations between the two farming systems (conventional vs. antibiotic free), including the range of scores assigned (0–2), the number of observations (Obs), the mean scores with standard deviations (SD) and the results of statistical analysis (*t*- and *p*-values). The presence of a statistically significant difference (*p* < 0.05) is indicated with an asterisk (*).

		Conventional			Antibiotic Free			*t*-Value	*p*-Value
Condition	Range	Obs	Mean	SD	Obs	Mean	SD		
HB	0–2	700	0.615714	0.627946	700	0.544286	0.611791	4.6708	<0.0001 *
FPD	0–2	700	1.095714	0.691857	700	1.007143	0.706565	2.3697	0.0179 *
Lameness	0–2	875	0.696	0.71526	875	0.485714	0.62971	6.5274	<0.0001 *

**Table 3:** Death rate in each pair of batches, including the difference calculated between the conventional and antibiotic-free batches. The average death rate in the two groups, with standard deviation (SD), is also reported.

Farm	Conventional Batch (%)	SD	Antibiotic-Free Batch (%)	SD	Difference
A(1)	1.95		1.80		0.15
B	2.07		3.27		−1.20
A(2)	3.00		2.46		0.54
C	1.23		1.23		0.00
D	3.11		2.42		0.69
E	2.21		2.66		−0.46
F	3.90		2.57		1.33
Mean	2.50	±0.89	2.34	±0.66	0.16
